# Is Hyperpigmentation on the First Day of Life Always Associated with IMAGe Syndrome?

**DOI:** 10.4274/jcrpe.1355

**Published:** 2014-12-05

**Authors:** Elif Özsu, Rahime Gül Yeşiltepe Mutlu, Olcay Işık, Filiz Mine Çizmecioğlu, Şükrü Hatun

**Affiliations:** 1 Kocaeli University Faculty of Medicine, Department of Pediatric Endocrinology, Kocaeli, Turkey; 2 Kocaeli University Faculty of Medicine, Department of Newborn Intensive Care Unit, Kocaeli, Turkey

**Keywords:** Intrauterine growth retardation, adrenal insufficiency, hypogonadism

## Abstract

IMAGe syndrome is an exceedingly rare condition first described in 1999. Components of the syndrome are intrauterine growth retardation (IUGR), metaphyseal dysplasia, congenital adrenal hypoplasia and genital anomalies. Cases generally present with life-threatening adrenal insufficiency in the neonatal period. Herein, we describe a patient with pronounced IUGR diagnosed with severe hyperpigmentation and adrenal insufficiency in the neonatal term in order to attract the attention to this rare entity.

## INTRODUCTION

IMAGe syndrome is a rare disease that is described with the major findings of intrauterine growth retardation (IUGR), metaphyseal dysplasia, congenital adrenal hypoplasia (CAH) and genitourinary abnormalities (in males). The diagnosis is confirmed in individuals with a heterozygous CDKN1C pathogenic variant in a specific domain of the maternally expressed allele. It is the only gene in which mutation is known to cause IMAGe syndrome. Here, we report a case presenting with severe generalized hyperpigmentation on the first day of life due to adrenal insufficiency and IUGR. A possibility of IMAGe syndrome was considered, but we could not find any mutations in the CDKN1C gene.

## CASE REPORT

**Figure 1:**
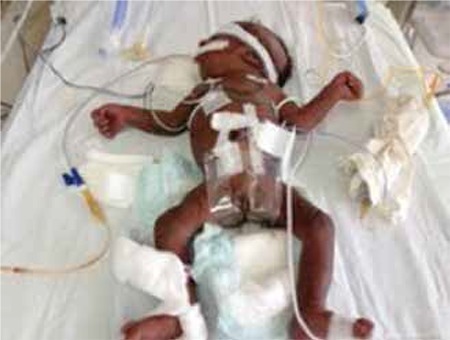
General appearance of the patient

**Figure 2:**
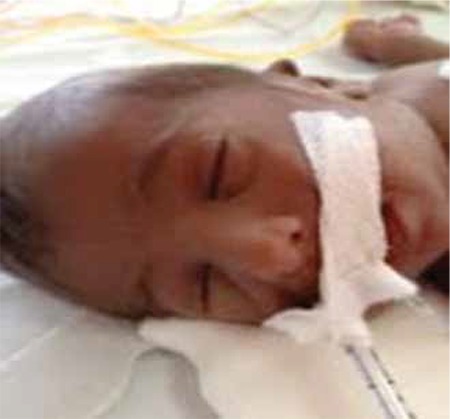
Facial appearance of the patient

**Figure 3:**
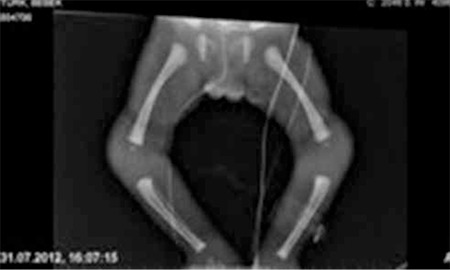
No skeletal abnormalities on X-ray image

This female patient was born at 39 weeks’ gestation to a 35-year-old father and a 28-year-old G3P3A2 mother. The parents were first-degree relatives. The infant, due to postnatal meconium aspiration, required intensive care including positive pressure ventilation. The baby’s weight at term was 2080 g (<10 p), her height was 46 cm (10-25 p) and her head circumference was 32 cm (10-25 p). Widespread hyperpigmentation was present over the entire body, including the mucosa (Figure 1). Facial appearance was dysmorphic. The ears were small and low-set bilaterally, the nasal bridge was flattened and retrognathia was present (Figure 2). The patient’s external genitalia were entirely female and no palpable gonads were detected bilaterally. On the second day of hospitalization, serum sodium level was 127 mEq/L and potassium level was 4 mEq/L. Severe hypoglycemia occurred twice during the observation period. On the third day, the patient’s adrenocorticotropic hormone level was >1250 pg/mL (n=10-60 pg/mL) and cortisol level was 0.3 µg/dL (n=1.7-14 µg/dL). Abdominal USG revealed absence of adrenal structures bilaterally and this was considered compatible with agenesis. Levels of adrenal products investigated to assess the adrenal functions were low (17 hydroxyprogesterone: 3 ng/mL; dehydroepiandrosterone sulfate: 15 µg/mL), while the aldosterone level at the time of the hyponatremia was 600 pg/mL (n=50-900 pg/mL). Investigation for possible accompanying hypogonadotropic hypogonadism revealed levels for follicle stimulating hormone of 0.4 u/mL, for luteinizing hormone <0.01 u/mL, for estradiol: 50 pg/mL and for testosterone (performed when the baby was 18 days old) 63 ng/dL, values much lower than appropriate for mini-puberty. The patient was started on an adrenal crisis protocol. A uterus was seen in ultrasonography and the karyogram was 46XX. Full bone scan imaging was performed and suspicious fractures were observed in the medial aspects of the bilateral femurs. The metaphysis and epiphysis were not yet affected (Figure 3). The patient’s condition worsened and multisystem failure developed. The patient was lost despite support treatment.

## DISCUSSION

IMAGe syndrome has a broad clinical spectrum. The etiology is not yet clear, although the syndrome itself has been well described. Early diagnosis is essential for the treatment of life-threatening adrenal insufficiency, since diagnosed cases generally present with findings of adrenal insufficiency in the first days of life. The syndrome was first described in 1999 (1), however, cases described as intrauterine development retardation, adrenal insufficiency, multiple congenital anomalies, growth hormone (GH) deficiency and pronounced motor retardation were reported before that date (2). Although the genetic transmission is autosomal recessive, some authors suggest the possibility of genomic imprinting with expression through maternal transmission involving an autosomal dominant gene (3).

Interestingly, DAX, SF, ACD and STAR mutations do not lead to the syndrome and in recent years, mutations occurring in the CDKN1C gene have been implicated in the disease. The gene responsible lies on 11p15 and contains a 17.2 megabase. Protein produced by this gene inhibits the cell cycle G0-G1 transition. Mutations proceeding with function acquisition lead to IMAGe syndrome, while inactivating mutations lead to Beckwith-Wiedemann syndrome. IMAGe syndrome has been shown to occur with maternal transmission. The pathology in this gene was identified by examination of seven cases in an Argentinean family (3).

IUGR was present in all patients reported to date (41/41) and this is generally accepted as a striking characteristic of the syndrome. On the other hand, results of GH stimulation tests and response to GH therapy have been shown to be variable.

The life-threatening components of the adrenal insufficiency syndrome generally develop in the neonatal period. Neonatologists in particular should consider this syndrome in case of sudden worsening in babies with IUGR and treatment must be initiated at once. Two cases not complying with this despite presenting with adrenal crisis in the first days of life have been reported (4). Our case demonstrated widespread hyperpigmentation with severe adrenal insufficiency. Despite the severe hyponatremia, potassium and aldosterone levels were within normal ranges. The clinical and laboratory spectrum of adrenal dysfunction is wide and cortisol and aldosterone values have been reported to remain within normal ranges at the time of adrenal crisis in some patients (5). The general condition of the patients has been found to improve with adrenal crisis treatment. Another endocrine involvement in these patients is GH deficiency. However, this has not been reported in all patients. Also, some cases with severe IUGR or small for gestational age and no GH deficiency have been started on GH therapy and a good response to GH has been reported.

Specific dysmorphic findings can also be present in patients with IMAGe syndrome; these include a protuberant forehead, low-set ears, frontal bossing, abnormal ears, short arms and legs, craniosynostosis, hypercalciuria, osteopenia, soft tissue/renal calcification, bone age retardation, short stature, a depressed nasal bridge and a short nose (6). Mild hypotelorism, the lateral parts of the eyebrows being low-set, a long filtrum, retrognathia and a high-arched palate and thin lower lip, bilateral low-set ears and pronounced occiput were also present in our patient.

In males in particular, genital anomalies such as hypospadias, micropenis, undescended testes and of varying severity may accompany the disease (4). Our patient was female. The external genitalia were completely female and no extra anomaly was detected despite the presence of dysmorphic findings.

Metaphyseal dysplasia can be one of the components of the IMAGe syndrome, although this involvement may not be easy to identify or may be quite mild. Longitudinal striations in the metaphysis may begin to be seen in the late infantile period and may progress with age. When there is no involvement in the metaphysis, the presence or absence of epiphyseal effects should be evaluated. There was no pronounced epiphyseal and metaphyseal involvement in our case. Narrow shoulders, osteoporosis, wide dysplastic metaphysis and a narrow growth plate are pathologies reported in previous cases, but there are no well-described findings to support the relationship of chondrodysplasia with this syndrome (2,3).

IMAGe syndrome was suspected in our patient due to IUGR, a history of sibling death, severe adrenal insufficiency and accompanying widespread hyperpigmentation. Despite treatment for adrenal crisis, the patient was unable to recover from sepsis and died. IMAGe syndrome is caused by gain-of-function missense mutations in the CDKN1C region encoding the PCNA-binding domain (amino acids 271-279) of the maternal allele, which cause loss of PCNA binding and pathogenic CDKN1C gain of function. We screened for mutation in exon 1 and 2 for the CDKNC1 gene, but no mutation was identified. We had no chance to perform genetic analyses in the siblings. Neither de novo mutation nor germline mosaicism has been reported, but both are possible, since CDKN1C mutation-negative IMAGe syndrome patients may have mutant genes that can be related to signal transduction pathway involving CDKN1C.

Hypercalcemia and hypercalciuria of unclear etiology and of variable degree can be encountered in patients with this syndrome. While the hypercalcemia may be a part of IMAGe syndrome itself, it may be secondary to sodium chloride supplementation, which is part of the treatment of the mineralocorticoid deficiency associated with adrenal insufficiency (3). Serum calcium and calcium homeostasis-related factors can be normal and dysregulated parathyroid hormone-related protein (PTHrP) and/or PTH/PTHrP receptor signalling might be relevant to skeletal abnormalities in patients with gain-of function mutations of CDKN1C. Such a possible signalling defect might also be relevant to the frequent occurrence of hypercalciuria in IMAGe syndrome (5). Our patient’s calcium levels were within the normal range.

Although rarely seen, adrenal insufficiency should be of primary consideration in patients born with IUGR who show widespread hyperpigmentation and who have a history of sibling death. Urgent hormone replacement treatment needs to be initiated in such cases.
